# Perianal Paget’s Disease: A Report of a Rare Case

**DOI:** 10.7759/cureus.83104

**Published:** 2025-04-27

**Authors:** Youssuf AlSuhaibani, Renad Y AlSuhaibani, Meshal A Alzakari, Fatemah Alhedaithy, Abdulaziz S Almodumeegh

**Affiliations:** 1 Department of Surgery, King Fahad Medical City, Riyadh, SAU; 2 College of Medicine, Imam Mohammed Ibn Saud Islamic University, Riyadh, SAU; 3 Department of Plastic and Reconstructive Surgery, Imam Mohammed Ibn Saud Islamic University, Riyadh, SAU

**Keywords:** atypical epidermal infiltrates, extramammary paget's disease (empd), multidisciplinary management, rare malignancy, reconstructive procedures

## Abstract

This case report discusses a 56-year-old Saudi female patient diagnosed with extramammary Paget's disease (EMPD) affecting the perianal region. Initially presenting with a one-year history of perianal itching and erythematous plaques, she underwent multiple evaluations, including a punch biopsy that revealed atypical epidermal infiltrates suggestive of EMPD or squamous cell carcinoma in situ. Comprehensive imaging and additional biopsies confirmed the diagnosis and assessed the extent of the disease. The patient underwent laparoscopic sigmoid colostomy and a wide local excision, followed by reconstructive procedures involving multidisciplinary surgical teams. Postoperative pathology confirmed perianal Paget's disease (PPD) with positive resection margins at the proximal vaginal area. The patient was discharged in stable condition and continues to receive follow-up care, highlighting the importance of multidisciplinary management in complex cases of perianal premalignancies and malignancies. Notably, to the best of our knowledge, no other case report has addressed a lesion of this size. Additionally, the initially uncertain location of the primary cancer lesion further distinguishes our case. Further studies are warranted to monitor for disease recurrence and evaluate the need for additional interventions.

## Introduction

Mammary and extramammary Paget's diseases both exhibit epidermal Paget's cells with a similar clinical presentation, but they are distinct in their anatomical locations and histogenesis [1]. Extramammary Paget's disease (EMPD) is a rare dermatologic disease that accounts for 6.5% of all cutaneous Paget disease [2]. Even rarer is secondary EMPD, which accounts for about 25% of cases [3]. EMPD is often found in areas with an abundance of apocrine sweat glands, most commonly the vulva, and predominantly occurs in females between 60 and 80 years of age [2]. Patients present clinically with well-demarcated erythematous plaques that persist despite using topical agents, and these lesions can progress to be erosive, ulcerated, scaly, or eczematous [2, 3]. Primary EMPD is a carcinoma in situ of apocrine origins, while secondary EMPD emerges as an extension to an underlying adnexal adenocarcinoma or visceral malignancies [2,3]. Primary and secondary EMPD can be differentiated through immunohistochemical staining [1]. Diagnosis and definitive treatment are frequently delayed because elderly patients often present at later stages and because nonspecific clinical findings usually lead to misdiagnosis [2]. Primary EMPD patients who receive appropriate treatment have an excellent prognosis, with reported recurrence rates ranging from 12% to 58%; thus, long-term follow-up is required [3]. Patients with invasive disease have a mortality rate between 13% and 18%, with a five-year survival rate of 72% [3].

EMPD is a rare condition characterized by non-squamous carcinoma cells in the skin, with less than 200 documented cases in the literature [4]. Given its rarity, there are currently no standardized protocols for the management or monitoring of perianal Paget's disease (PPD). Its treatment is challenging for clinicians due to its rarity; thus, ongoing surveillance is necessary in such cases. This case study aims to discuss a unique presentation of PPD, exploring the challenges and decision-making processes encountered during the clinical journey of the patient. The objective is to present a thorough case analysis that contributes valuable insights into EMPD management.

## Case presentation

Patient information

A 56-year-old Saudi female patient with no significant chronic illnesses presented with a 20-year history of bipolar disorder, which had been effectively managed with medication. She had no notable family history of malignancy.

History of present illness

She presented with a one-year history of perianal itching, accompanied by an erythematous plaque in the perianal region. Initial treatment at a dermatology clinic was ineffective, prompting her referral to King Fahad Medical City, Riyadh, Saudi Arabia, along with a punch biopsy report for further assessment and management.

Diagnostic assessment

**Figure 1:**
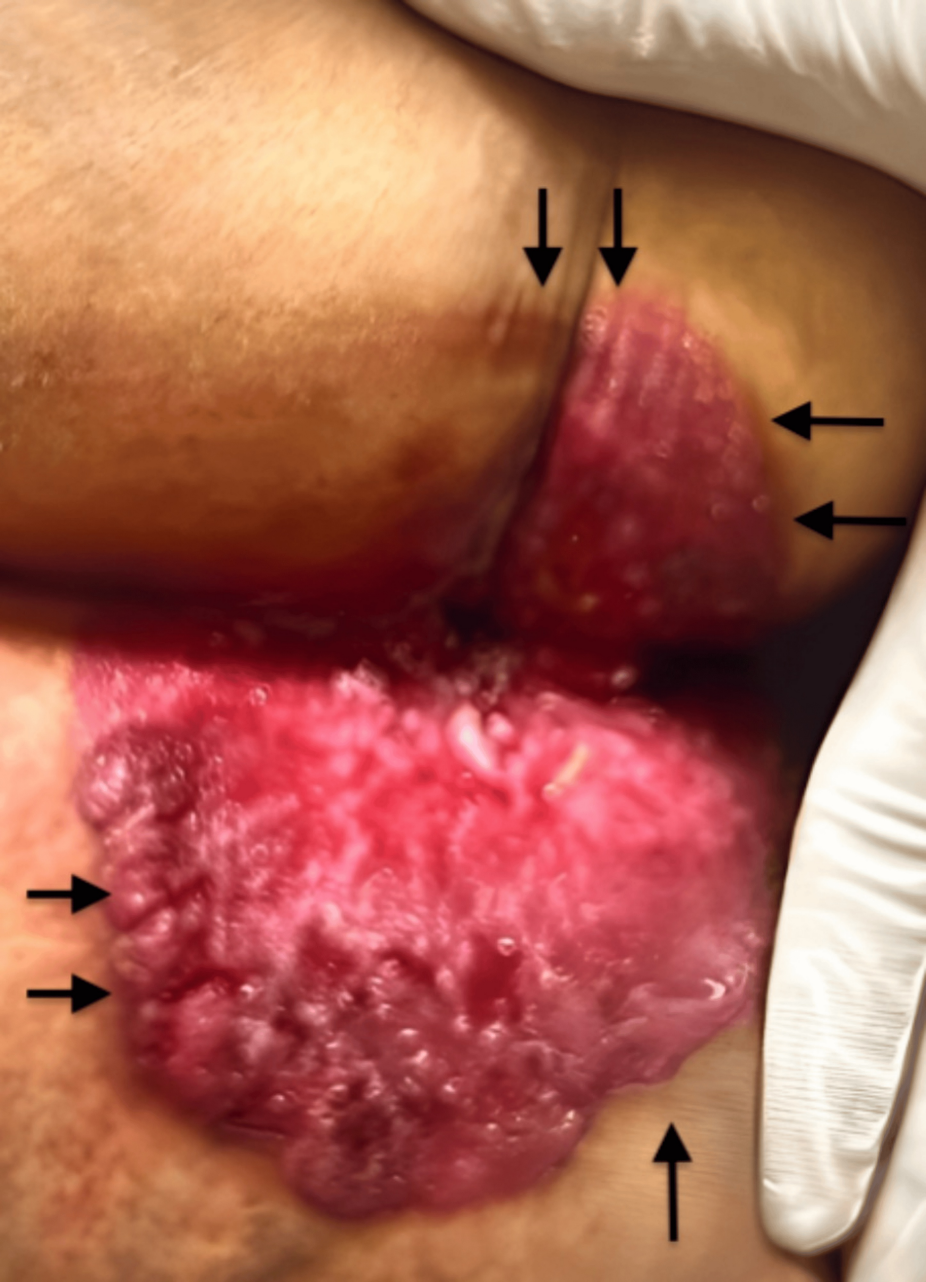
Perianal erythematous plaque characterized by significant erythema and irregular surface.

During the physical examination, circumferential perianal redness and ulceration measured 14 x 12 cm (Figure 1). This image illustrates the lesion of PPD, as seen immediately before surgical excision.

**Figure 2:**
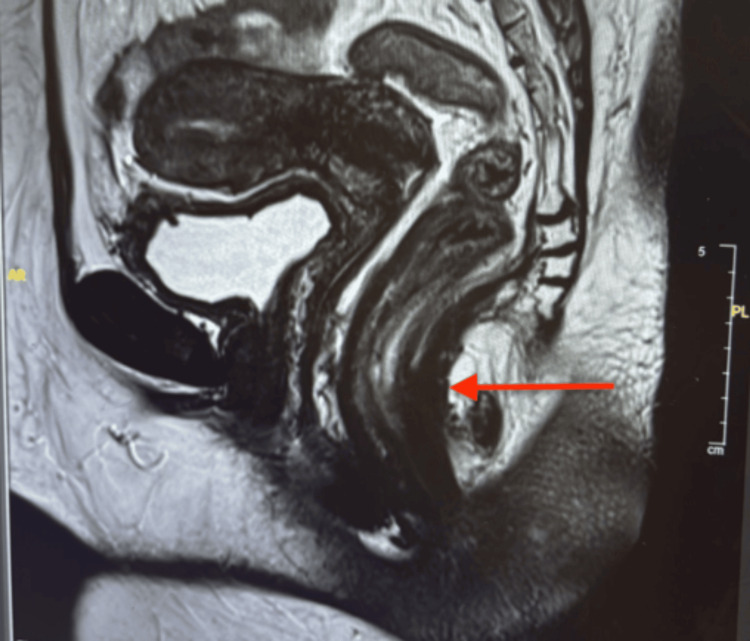
Pre-surgical MRI of the pelvis shows that the perianal region, anus, and rectum appear normal, with no evidence of wall thickening or masses (arrow).

**Figure 3:**
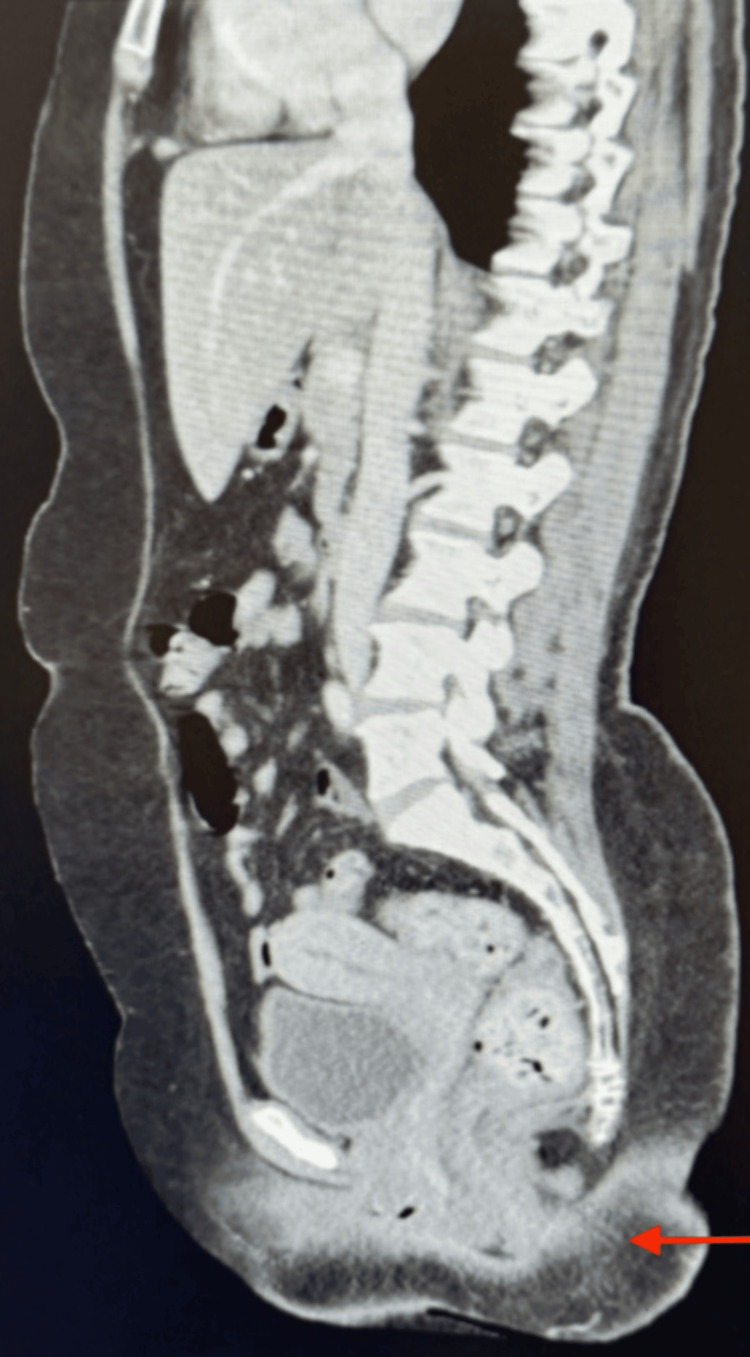
Pre-surgical enhanced CT scan of the abdomen and pelvis with IV contrast shows no abdominopelvic metastasis (arrow).

Imaging studies, including MRI and CT scans of the abdomen, pelvis, and chest, revealed no evidence of metastases (Figures 2, 3). A colonoscopy showed edematous mucosa. The punch biopsy demonstrated an atypical epidermal infiltrate, with differential diagnoses including EMPD and squamous cell carcinoma in situ.

Tissue pathology findings

The pathology examination of the vulva, following the modified radical vulvectomy, confirmed a diagnosis of EMPD. The margins were positive at the proximal vaginal margin but negative at the deep resection margin. Importantly, there was no stromal invasion, and foreign body-type granulomatous inflammation was observed. In the cervix, the excision of a polyp resulted in a diagnosis of a benign endocervical polyp, with findings negative for dysplasia or malignancy. The rectal tissue obtained from the low anterior resection also indicated EMPD, with margins testing negative for Paget's disease. Examination revealed no stromal invasion and identified a hyperplastic polyp in the colonic mucosa. Nine reactive lymph nodes were assessed, all negative for malignancy, and foreign body-type granulomatous inflammation was noted.

Therapeutic intervention

The patient was admitted on September 5, 2023, for examination under anesthesia (EUA), biopsy, and laparoscopic sigmoid colostomy. The surgery was performed without complications, and her postoperative recovery was smooth. Multiple punch biopsies from various locations around the anus confirmed a diagnosis of secondary EMPD (Table 1).

**Table 1:** Summary of final diagnoses and comments from biopsy reports of perianal lesions Confirmatory immunostaining with appropriate controls performed on this case revealed that the tumor cells are positive for CDX2 and MUC2 (focal) but negative for CK5/6, S100, p16, and GCDFP15. The overall findings were in support of Paget's disease diagnosis and in favor of lower GI primary, among others.

Biopsy	Comments and diagnosis
1. Anus, left anterolateral close to the anus	Consistent with secondary extramammary Paget's disease
2. Anus, left anterolateral away from the anus	
3. Anus, left posterolateral away from the anus	
4. Anus, left posterolateral close to the anus	
5. Anus, right anterolateral close to the anus	
6. Anus, right anterolateral away from the anus	Polypoid fragment of tissue with perianal glands; no surface epithelium available for evaluation; negative for malignancy
7. Anus, right posterolateral away from the anus	Consistent with secondary extramammary Paget's disease
8. Anus, right anterolateral away from the anus	Consistent with secondary extramammary Paget's disease

The patient was readmitted on November 27, 2023, for a wide local excision of the perianal disease, which took place on November 28, 2023. The patient remained vitally stable and was doing well postoperatively. Tissue pathology and cultures were sent for further analysis. Examination of the specimen revealed epithelioid tumor cells exhibiting nested and pagetoid spread within the epidermis and skin appendages. These cells displayed abundant pale cytoplasm, enlarged pleomorphic nuclei, and prominent nucleoli.

Immunostaining results indicated positivity for CK7 (partial), CK20, CDX2, and CAM5.2, while being negative for GCDFP15, S100, SOX10, GATA3, PAX8, and CK5/6. Beta-catenin staining exhibited a wild-type pattern with no nuclear staining (Table 1). Importantly, both the anal verge and vaginal introitus resection margins were positive for Paget's disease, as confirmed by immunohistochemical staining, while the deep resection margin was negative. 

**Figure 4:**
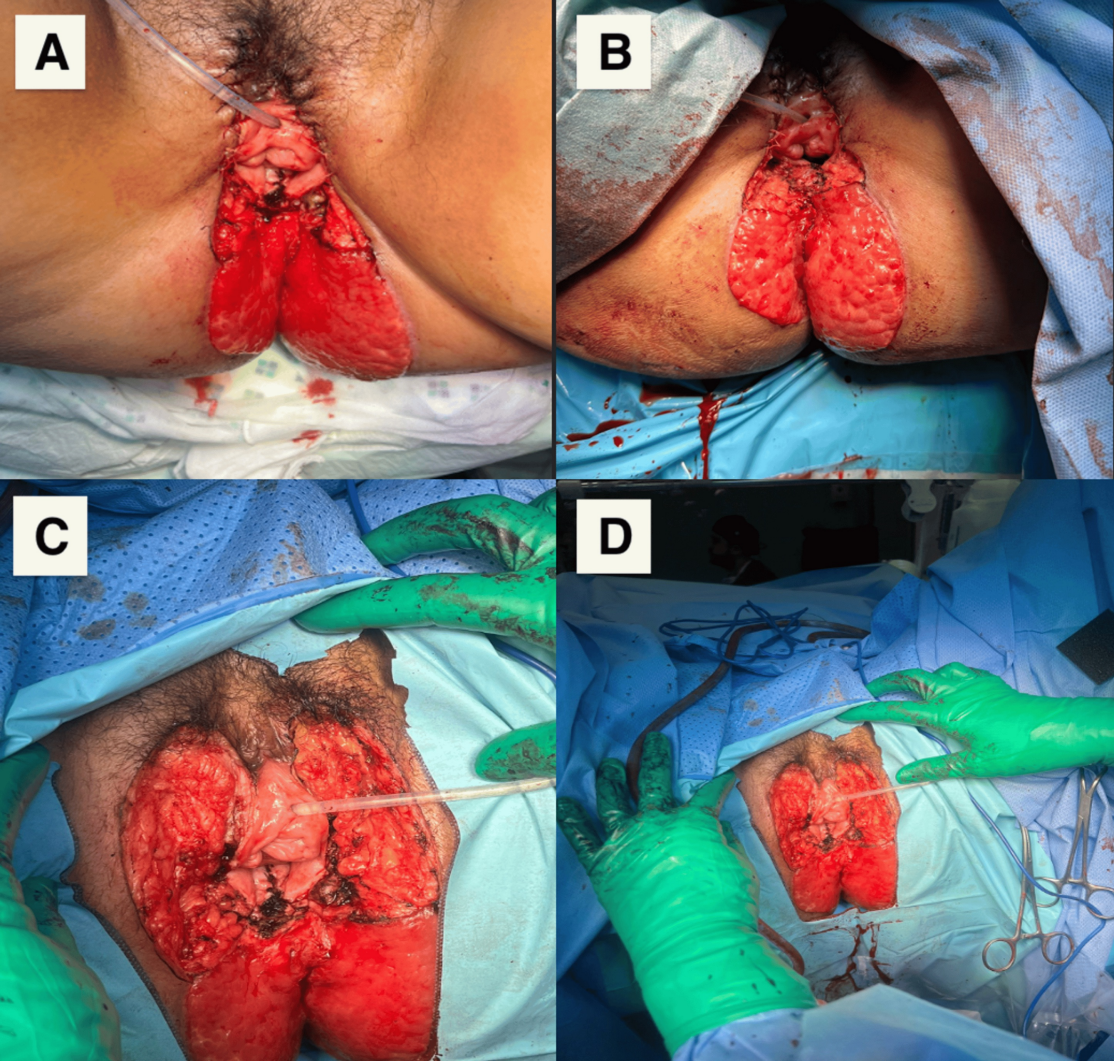
Laparoscopic abdominal perineal resection with modified radical vulvectomy and resection of a portion of the posterior vagina with reconstruction of the perineal defect by bilateral transposition flaps (A) Picture of the perineum after the first surgery (after excision of the lesion) with preservation of the anal canal and the vagina; (B) Picture of the perineum after the first surgery (after excision of the lesion with preservation of the anal canal and the vagina) two weeks later; (C) Picture of the perineum after the second surgery (after abdominal perineal resection and complete excision of the lesion, including the posterior wall of the vagina); (D) Zoom out to a wider angle to capture a broader view of the C shot.

A collaborative treatment plan was established involving general surgery, plastic surgery, and gynecologic oncology. The team agreed on a laparoscopic abdominal perineal resection, modified radical vulvectomy, resection of a portion of the posterior vagina, and reconstruction of the perineal defect using bilateral transposition flaps (Figure 4). The images illustrate the procedure, showcasing the laparoscopic abdominal perineal resection, including vulvectomy, posterior vaginal resection, and perineal defect reconstruction using flaps.

Postoperative summary

**Figure 5:**
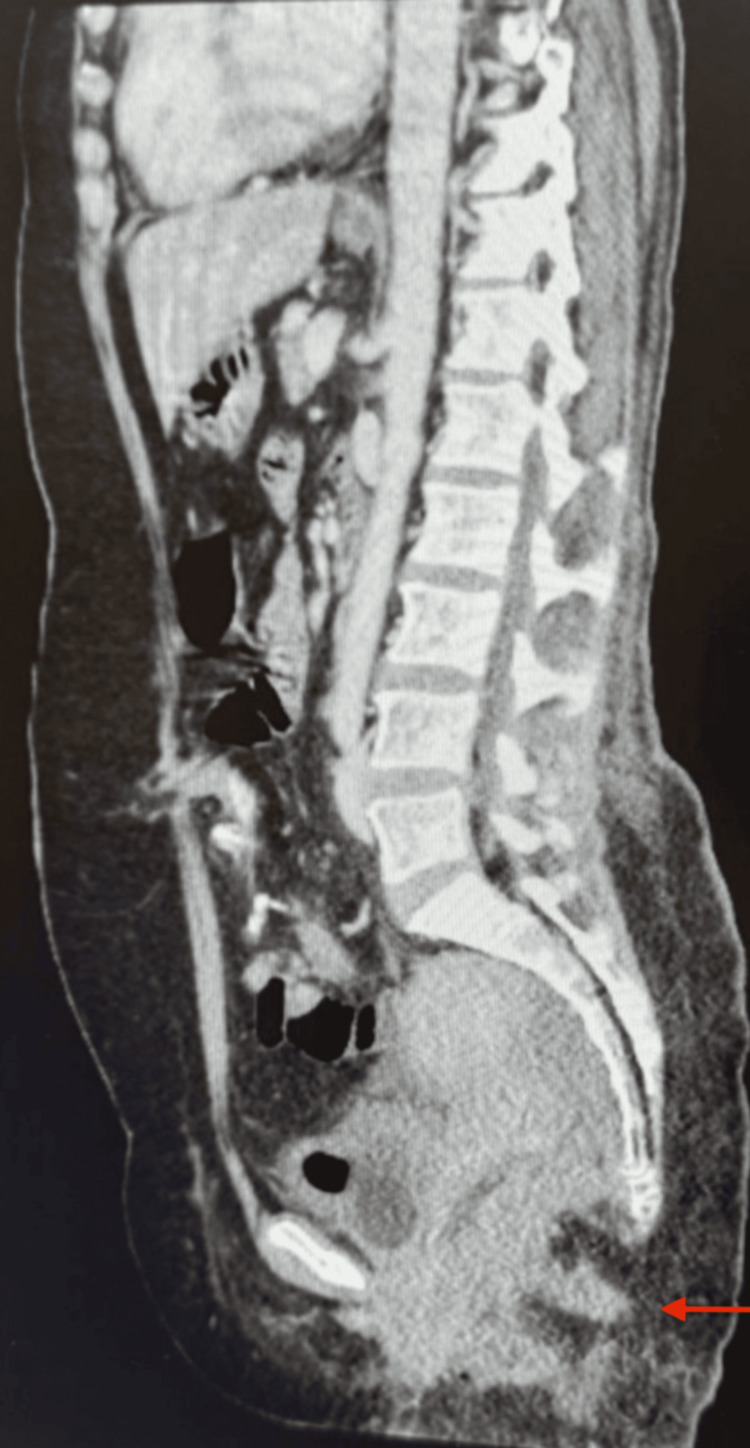
Post-surgery CT of the abdomen and pelvis with IV contrast shows no definitive fluid collection, no bowel obstruction, and unremarkable abdominal organs, with no significant lymph nodes or any vascular concern.

The surgery was successful, with no complications, and the patient remained vitally stable throughout the postoperative period. A CT scan on December 23, 2023, revealed no definitive fluid collections, although mild bilateral pleural effusions were noted, as illustrated in Figure 5. The patient was discharged on December 28, 2023, and follow-up appointments were scheduled one week later in the outpatient department (OPD) for plastic surgery, gynecology, and colorectal surgery.

**Figure 6:**
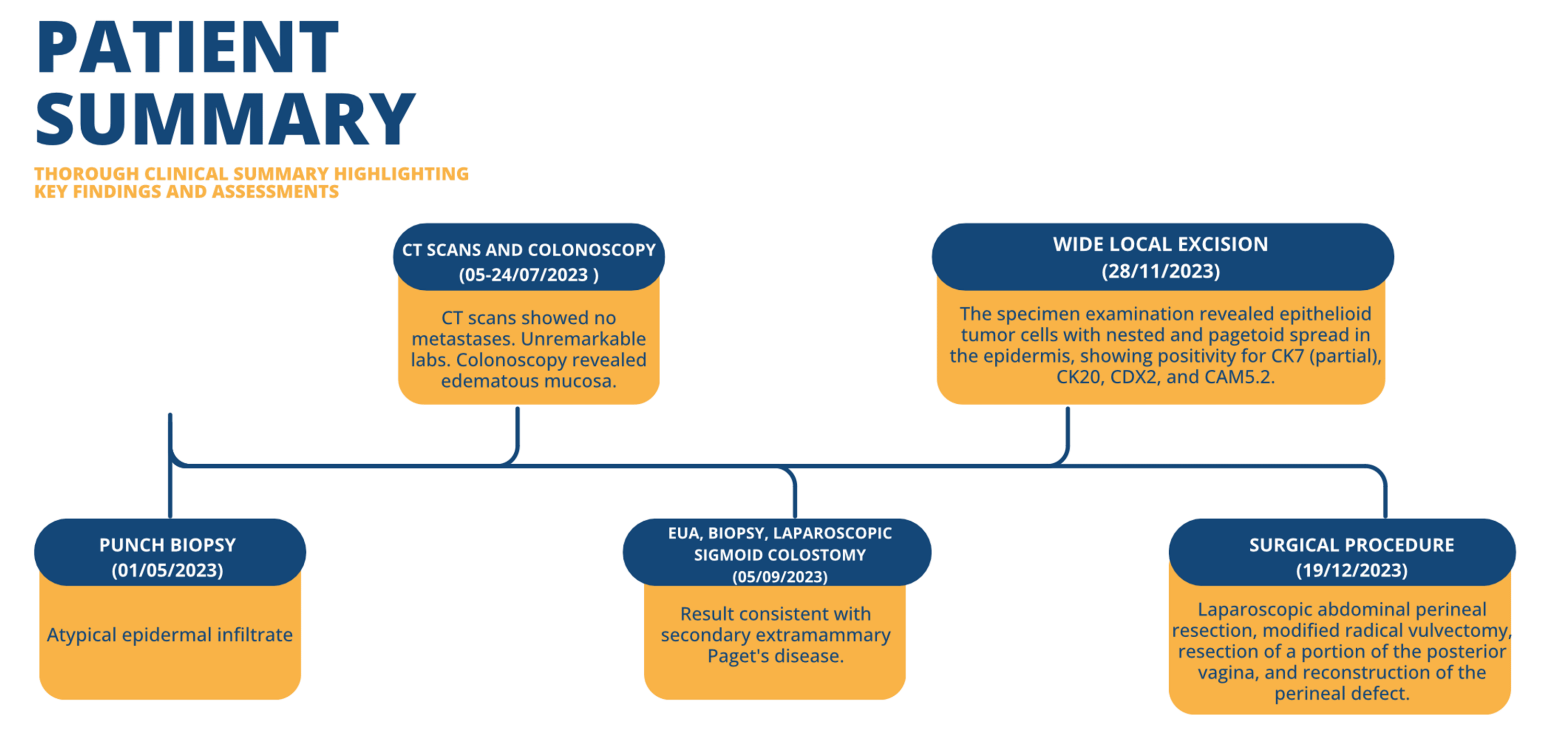
The patient's case summary EUA: examination under anesthesia

During the OPD visits, the patient reported feeling well; however, some perineal flap excoriation and a small wound gaping were observed, leading to recommendations for sitz baths. Healing was excellent during the plastic surgery follow-up, despite some vaginal discharge. Laboratory results were unremarkable at the time of discharge. Patient case summary presented in Figure 6.

## Discussion

PPD is an exceedingly rare manifestation of EMPD, with few cases reported in the literature [5]. This case presents a 56-year-old Saudi female patient diagnosed with secondary EMPD, a rare and challenging condition that typically affects the genital, perianal, and axillary regions [6, 7]. The patient’s history of bipolar disorder was not directly related to her presenting symptoms, and she had no significant chronic illnesses or family history of malignancy. Her condition started with a year-long history of perianal itching and erythematous plaques, which was initially misdiagnosed and treated ineffectively at a dermatology clinic. The eventual referral to a tertiary care center, along with the punch biopsy results, led to further investigation and diagnosis of EMPD.

PPD is often misdiagnosed due to its nonspecific clinical presentation, which can mimic benign dermatological conditions such as eczema, psoriasis, or fungal infections [8-10]. In this case, the patient’s symptoms were initially treated with topical agents without improvement, leading to a delay in diagnosis. The definitive diagnosis was made through punch biopsy, which revealed atypical epidermal infiltrates. Immunohistochemical staining was pivotal in differentiating primary from secondary EMPD. The tumor cells exhibited positivity for CK7 (partial), CK20, CDX2, and CAM5.2, while being negative for GCDFP15, S100, SOX10, GATA3, PAX8, and CK5/6. This staining pattern is consistent with secondary EMPD, often associated with underlying colorectal or genitourinary malignancies [11, 12]. However, no underlying malignancy was identified in this patient, which is unusual and adds to the diagnostic complexity of PPD.

The gold standard treatment for EMPD is surgical excision with clear margins [13]. However, achieving negative margins in perianal lesions is particularly challenging due to the anatomical complexity and functional considerations of the perineal region. In this case, the patient underwent a staged surgical approach, beginning with a laparoscopic sigmoid colostomy to divert fecal flow and facilitate wound healing. This was followed by wide local excision and, ultimately, a radical resection involving laparoscopic abdominal perineal resection, modified radical vulvectomy, and posterior vaginal resection. The use of bilateral transposition flaps for perineal reconstruction clarifies the importance of plastic surgery in achieving both functional and cosmetic outcomes [14]. Histological examination of the excised tissues showed a clear diagnosis of EMPD with no stromal invasion and foreign body-type granulomatous inflammation, suggesting a localized disease with no metastatic spread. However, during these efforts, the proximal vaginal margin remained positive for Paget's disease, highlighting the difficulty of achieving complete resection in extensive cases. Positive margins are a known risk factor for recurrence, which occurs in 12% to 58% of cases [15]. Long-term follow-up is essential to monitor for recurrence and manage any subsequent interventions. 

The unique aspect of this case was the size of the lesion (14 x 12 cm), which is larger than most reported cases of PPD. Additionally, the initially uncertain location of the primary cancer lesion adds to the uniqueness of this presentation. The absence of an underlying malignancy despite the immunohistochemical profile suggestive of secondary EMPD further distinguishes this case from others in the literature. These factors highlight the need for individualized treatment plans and the importance of a multidisciplinary approach in managing complex cases of PPD.

The prognosis for EMPD depends on whether the disease is primary or secondary and the presence of invasive components [16]. Primary EMPD confined to the epidermis has an excellent prognosis, while secondary EMPD or invasive disease is associated with higher mortality rates [17]. In this case, the absence of stromal invasion and underlying malignancy suggests a favorable prognosis. The postoperative recovery was smooth, and the patient showed positive progress, with some minor wound-healing issues noted, which were managed conservatively. The follow-up appointments revealed good overall healing, though the patient required additional care for perineal flap excoriation and minor wound gaping. Importantly, no systemic complications were reported, and the patient’s lab results were unremarkable at discharge, suggesting that the disease was localized and there was no evidence of metastatic spread. However, the positive resection margins necessitate close follow-up to detect and manage recurrence promptly. 

## Conclusions

This case report contributes to the growing body of literature on PPD, emphasizing the importance of early diagnosis, comprehensive diagnostic evaluations, and multidisciplinary management strategies. The rarity of PPD poses significant challenges, but through detailed case analysis and sharing of clinical experiences, we can enhance our understanding and improve the outcomes for future patients diagnosed with this rare condition.
